# Conservation of Endophyte Bacterial Community Structure Across Two *Panicum* Grass Species

**DOI:** 10.3389/fmicb.2019.02181

**Published:** 2019-09-27

**Authors:** Esther Singer, Jason Bonnette, Tanja Woyke, Thomas E. Juenger

**Affiliations:** ^1^US Department of Energy, Joint Genome Institute, Walnut Creek, CA, United States; ^2^Department of Integrative Biology, University of Texas at Austin, Austin, TX, United States

**Keywords:** plant microbiome, *Panicum*, rhizosphere, root endosphere, core microbiome, biofuel

## Abstract

*Panicum* represents a large genus of many North American prairie grass species. These include switchgrass (*Panicum virgatum*), a biofuel crop candidate with wide geographic range, as well as *Panicum hallii*, a close relative to switchgrass, which serves as a model system for the study of *Panicum* genetics due to its diploid genome and short growth cycles. For the advancement of switchgrass as a biofuel crop, it is essential to understand host microbiome interactions, which can be impacted by plant genetics and environmental factors inducing ecotype-specific phenotypic traits. We here compared rhizosphere and root endosphere bacterial communities of upland and lowland *P. virgatum* and *P. hallii* genotypes planted at two sites in Texas. Our analysis shows that sampling site predominantly contributed to bacterial community variance in the rhizosphere, however, impacted root endosphere bacterial communities much less. Instead we observed a relatively large core endophytic microbiome dominated by ubiquitously root-colonizing bacterial genera *Streptomyces*, *Pseudomonas*, and *Bradyrhizobium*. Endosphere communities displayed comparable diversity and conserved community structures across genotypes of both *Panicum* species. Functional insights into interactions between *P. hallii* and its root endophyte microbiome could hence inform testable hypotheses that are relevant for the improvement of switchgrass as a biofuel crop.

## Introduction

Most land plants grow in intimate association with a complex microbiota. Microorganisms in the belowground plant compartments can live on the inside (endophytes), on the outside (epiphytes), or closely associated with the plant (Philippot et al., 2013). A number of recent studies in various plants showed that root microbiome compositions are impacted by planting sites/soil types (Lundberg et al., 2012), plant species as well as among genotypes within a single species (Schweitzer et al., 2008; Bever et al., 2012; Lundberg et al., 2012; Wagner et al., 2016a). While differentially abundant microorganisms may be linked to, e.g., cultivar-specific recruitment factors, such as host genetic regions, core microbiomes allow insights into inherited microbial players that may have evolved together with their hosts across genotypes, species, and even sites (Whitham et al., 2006). Plant microbiomes can serve various plant growth supporting functions, for example they can act as protectants against phytopathogens, improve growth through production of phytohormones, and help plants withstand heat, salt, and drought (Yeoh et al., 2017). The plant in turn cultivates its microbiomes by adjusting the soil pH, reducing competition for beneficial microbes, and providing carbon sources, mostly in the form of carbon-rich rhizodeposits (Firáková et al., 2007; Mendes et al., 2011). The identification and characterization of plant growth-promoting bacteria hence provides a lever to enhance plant performance for sustainable agriculture (Sturz and Nowak, 2000; Nelson, 2004).

Switchgrass (*Panicum virgatum*) is a perennial warm season grass native to North America that includes a large variety of genotypes allowing it to adapt to various climatic conditions and soil types. Switchgrass is a favored biofuel candidate for a number of reasons: it is a productive long-lived perennial crop with high biomass production potential, efficient water use, and relatively low demand for nutritional inputs and agrochemicals. It has high carbon sequestration potential and is well adapted to marginal soils (Marschner et al., 1986; Dennis et al., 2010). However, genetic improvements and widespread commercial use have partly been challenged by its genetic complexity. This also renders studies such as the identification and correlation of plant growth-promoting bacteria (PGPB) to gene regions and genetic expression associated with stress response challenging and labor intensive. *Panicum hallii* is a close relative of agronomic switchgrass. In contrast to the large and variable genome of switchgrass (McLaughlin and Adams Kszos, 2005; Sanderson et al., 2006), *P. hallii* has a diploid genome, for which next-generation sequencing is more cost-effective, genetic mapping is likely to be more powerful due to simple genetics, and transgenic experiments are likely to be easier because of the inbred and diploid features of *P. hallii*. Hence *P. hallii* represents a valuable resource for the optimization of genomic tools for *P. virgatum*. Both *P. virgatum* and *P. hallii* include two major ecotypes found in xeric or mesic habitats. Host ecotype-differentiation has been observed to impact and be impacted by the structure and function of the associated microbiomes (Bulgarelli et al., 2012; Laine et al., 2014). Few studies exist on microbial communities associated with switchgrass (Allgaier et al., 2010; Gladden et al., 2011; D’haeseleer et al., 2013; Gagne-Bourgue et al., 2013; Xia et al., 2013; Piao et al., 2014; He et al., 2017; Chen et al., 2019), and only fungal sequence data have been collected on *P. hallii* varieties (Giauque and Hawkes, 2016). In this study, we compared root endophytic and rhizosphere microbial community structure and diversity among plants representing both ecotypes in *P. virgatum* and *P. hallii*. We report microbial community members distinct between and conserved among rhizosphere and root endosphere, planting sites, and *Panicum* species. The aim of this study is to evaluate opportunities of using *P. hallii* as a model plant for microbiome studies that can then be tested on and applied to switchgrass.

## Materials and Methods

### Switchgrass Plants

**Figure 1:**
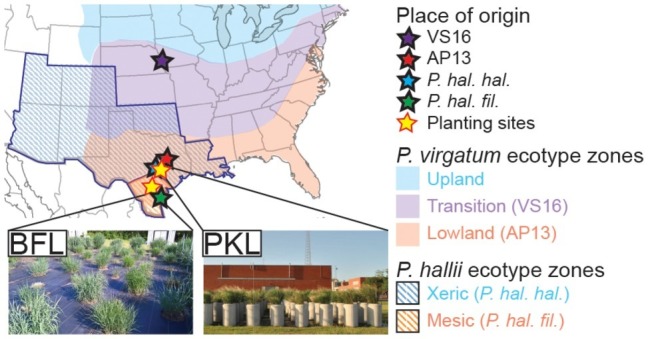
Description of sample origin, natural distribution of *Panicum* ecotypes and planting sites. *P. virgatum and P. hallii* plants were planted at two locations (PKL and BFL field sites) in Texas (yellow stars). Sites of origin for each ecotype are denoted with stars of distinct colors (blue ~ uplands, red ~ lowlands). Colored regions show where *Panicum virgatum* ecotypes (blue, purple and red) as well as *Panicum hallii* ecotypes (blue and red stripes) naturally occur.

Switchgrass (*Panicum virgatum*) plants selected for this study include clonal divisions of two ecotypes: Alamo-AP13 (the lowland switchgrass reference genome clone, a selection from the Alamo cultivar collected along the Frio River in Live Oak County, TX) and Summer-VS16 (the upland switchgrass reference genome clone, a selection from the Summer cultivar collected from near Nebraska City, NE). *Panicum hallii* plants included in this study are represented by clonal divisions of two ecotypes: *P. hallii* var. *hallii* [the upland reference genome clone, a selection from the Wild Flower Center in Austin, TX (30°48′11.5″N, 97°53′55.1″W)] and *P. hallii var. filipes* [the lowland reference genome clone, a selection from the Botanical Garden in Corpus Christy, TX (27°39′10.3″N, 97°24′24.8″W)] (Lovell et al., 2018). Clonally propagated switchgrass seedlings and *P. hallii* seedlings grown from seed (Figure 1A, Supplementary Table S1) were planted in a random block design in April 2013 outdoors in concrete cylinders (2 ft. diameter by 4 ft. height) containing Ranch Rose potting soil (Geo Growers, Austin, TX) at the Pickle Research Center facility of the University of Texas in Austin, TX (PKL) (30°23′11.8″N 97°43′36.8″W) and in natural soil at the Brackenridge Field Lab of the University of Texas in Austin, TX (BFL) (30°17′2.4″N 97°46′40.8″W) (Supplementary Table S1). Plants were irrigated amply and allowed to establish during the 2013 growing season. Each genotype at each site was represented by five replicate plants in this study totaling 40 plants (4 genotypes × 2 sites × 5 biological replicates = 40) (Supplementary Table S2).

### Sample Collection, Preparation, and DNA Extraction

Soil, rhizosphere (RS), and root endosphere (RE) samples were collected in August 2015. From each surveyed plant, soil cores with disposable sterile insert sleeves were used to collect root and soil material to a depth of 1 m. Bulk soil was taken at BFL from random locations within field site, and at PKL from within the cylinders. Soil and root samples were consolidated across the entire depth of 1 m. RS samples were defined as soil attached within approximately 1 mm of roots after vigorously shaking to remove bulk soil from root systems. RS samples were obtained by washing 4 g of root material with 45 ml of buffer (0.1× PBS buffer with 0.1% Triton X-100) on a tabletop shaker (200 rpm for 15 min). Root wash solutions were stored at −80°C with 10% glycerol and later vacuum-filtered onto 0.2-μm GTTP filter membranes (Whatman, Maidstone, UK). Half of the filter membranes were processed with the MoBio™ Power Soil DNA extraction kit (Carlsbad, CA, USA) following the manufacturer’s guidelines. For RE samples, roots were washed with 35 ml of tap water in a 50-ml tube, sterilized with 35 ml of 3% sodium hypochlorite solution while gently shaking for 2 min, rinsed with 35 ml of sterile MilliQ water twice while gently shaking, ground with liquid nitrogen and frozen at −80°C. We used 0.25 g of each RE sample for DNA extraction with the MoBio™ Power Soil DNA extraction kit (Carlsbad, CA, USA) following the manufacturer’s guidelines. RE samples include root endosphere and rhizoplane microorganisms. DNA concentrations were quantified using a Pico Green assay (Thermo Fisher, Waltham, MA).

### Polymerase Chain Reaction Amplification and Sequencing

For universal amplification of the V4 region of the 16S rRNA gene (V4 iTags), we used forward primer 515 F (5′-GTGCCAGCMGCCGCGGTAA-3′) and reverse primer 806 R (5′-GGACTACHVGGGTTCTAAT-3′) containing a variable 12-bp barcode sequence (Caporaso et al., 2012) in combination with PNA clamps to block plant genomic DNA during amplification in endosphere samples as described (Lundberg et al., 2013). iTag sequencing was performed according to JGI’s standard procedures^1^: V4 amplicons were diluted to 10 nM, quantified by quantitative PCR, pooled at 92 samples per run, and sequenced via 4,000 pM of pool DNA spiked with 5% PhiX on the Illumina MiSeq platform (reagent kit v.3; Illumina, Inc., San Diego, CA) (Keesing et al., 2010; Berendsen et al., 2012; Shreiner et al., 2015; Tremblay et al., 2015). Soil samples from the BFL site failed twice either during PCR amplification or during sequencing, which we hypothesize is due to the high clay content that could have inhibited PCR and/or due to the relatively low biomass in that soil. For consistency, we excluded all soil samples from PKL and BFL from the following analysis. A count overview of successfully extracted, amplified, and sequenced samples is displayed in Supplementary Table S2.

### Operational Taxonomic Unit Filtering and Normalization

V4 iTag sequences were quality screened, demultiplexed, and clustered for operational taxonomic unit (OTU) analysis using iTagger v1.2 (Tremblay et al., 2015), which includes QC (LEN_MEAN = 292, LEN_STDEV = 20, TRIM5 = 0, TRIM3 = 0), contamination filtering (KMER = 21, STEP = 1, CUTOFF = 1), primer removal (ERROR + RATE = 0.15, MIN_OVERLAP = 18), paired-end read merging (MIN_OVERLAP = 20, MAX_MISMATCH = 0.3), read clustering (MIN_LIB_SIZE = 500, MAX_RADIUS = 3), OTU filtering and chimera removal (MIN_NORM_SIZE = 10, MIN_NUM_LIBS = 2), and classification using the Greengenes database (2013-04-25) (MIN_WORDS = 120, LEVEL = kingdom, CUTOFF = 0.5) (Desantis et al., 2006). iTag sequences were grouped into operational taxonomic unit (OTU) clusters using a 97% identity threshold. Datasets were rarefied to 10,000 reads per sample because this rarefaction level was found to include a representative fraction of the microbiome (Supplementary Figure S2) and retained most samples (Supplementary Figure S3). We are also providing factor contribution and core microbiome analyses on non-rarefied OTU tables in the Supplementary Material in order to validate our trends observed with the rarefied OTU tables. To reduce low-abundance and spurious OTUs, OTUs were kept only if they had at least five reads in at least three samples using QIIME v.1.9.1 (Caporaso et al., 2010). We identified 7,556 OTUs in the RS and 2,299 OTUs in the RE with ≥5 reads in respective plant niches. Sequence data, mapping files, and iTagger config file are publicly available at https://portal.nersc.gov/dna/metagenome/assemblies/Panicum/.

### Statistical Analyses

**Figure 2:**
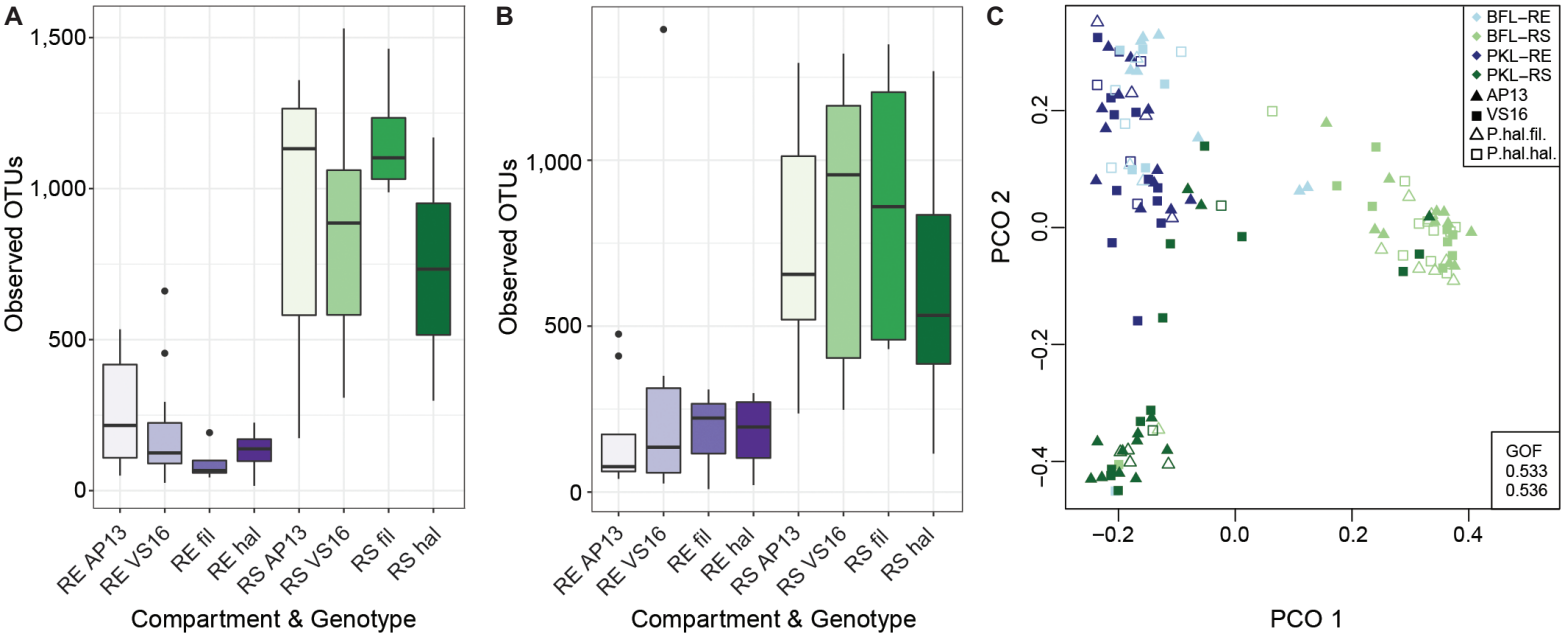
Alpha Diversity of root and soil microbiomes at PKL **(A)** and at BFL **(B)** and Principal Coordinate Analysis of RS and RE samples from both sites **(C)**. Colors denote plant compartment, shapes in PCO plot show ecotype. Panicum species and compartments are abbreviated as follows: *P. hal.* var. *fil.* ~ filipes, *P. hal. hal. ~ hallii*, *P. virgatum* AP13 ~ AP13, *P. virgatum* VS16 ~ VS16; Rhizosphere ~ RS, Root Endosphere ~ RE. GOF = Goodness of fit.

**Figure 3:**
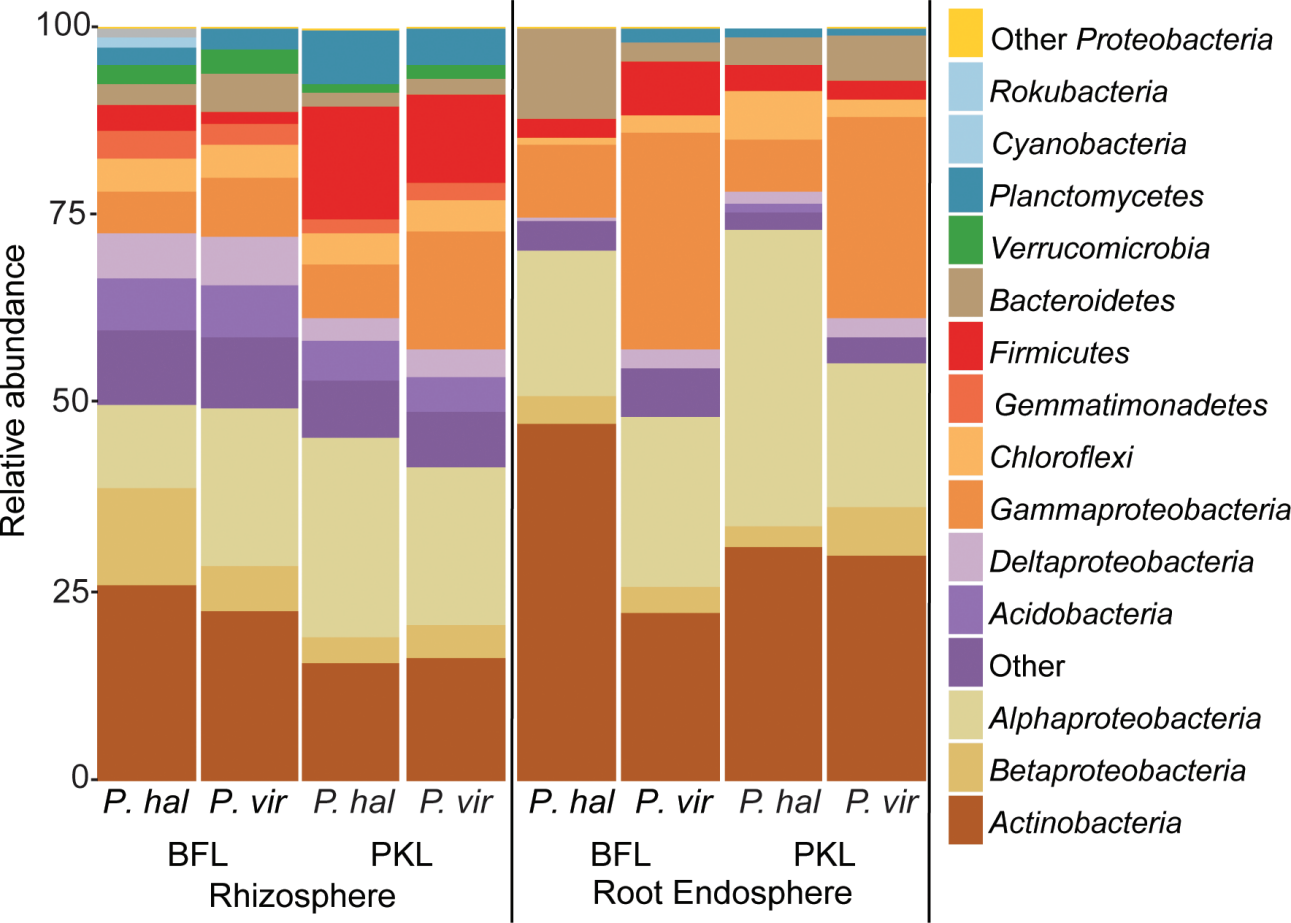
Relative abundance of bacterial phyla and *proteobacterial* classes in rhizosphere and root endosphere from *P. hallii* (*P. hal*) and *P. virgatum (P. vir)* at planting sites BFL and PKL. Taxonomic groups with ≥1% relative abundance are listed; phyla at <1% relative abundance are summarized as “Other.”

Alpha-diversity analyses – Observed OTUs (Figures 2A,B), Chao1 richness (Supplementary Figure S4) and Shannon’s (*H*′) index (Supplementary Figure S5) – were performed in QIIME v.1.9.1 (Caporaso et al., 2010). Factor contributions to % community variance as explained by factors planting site, plant compartment, and plant ecotype were assessed based on weighted and unweighted UniFrac distances of rarefied (Table 1) and non-rarefied (Supplementary Table S3) OTU tables, and computed in QIIME v.1.9.1 (Caporaso et al., 2010) and using the *adonis* function with 999 permutations as part of the Vegan package v. 2.4-6 (Oksanen et al., 2018) in R v. 3.4.3 (R Development Core Team, 2008). Tree construction for UniFrac calculations was achieved by aligning OTU sequences with MAFFT v. 7.221 (Katoh et al., 2002) and branch length calculation using FastTree 2 (Price et al., 2010). Principal Coordinate Analysis to show grouping of samples by plant compartment was carried out in R (R Development Core Team, 2008) based on Bray-Curtis distances and using the *vegdist* function of the Vegan package using the rarefied OTU table. Beta-diversity was calculated using unweighted UniFrac (beta_diversity.py) and community variance by factor was evaluated using the Kruskal-Wallis test (group_significance.py), both performed in QIIME v.1.9.1 (Caporaso et al., 2010). Relative abundances of phyla are represented by averages across sample groups. Phyla with <1% relative abundance were consolidated as “Other.” Core microbiome OTUs were defined as present in 80% of all samples and were computed in QIIME v.1.9.1 on rarefied (Figure 3) and non-rarefied (Supplementary Figure S6) OTU tables for comparison (compute_core_microbiome.py).

**Table 1:** Percent bacterial community variance according to weighted and unweighted Unifrac distance explained by factors for all samples (A), samples grouped by *Panicum species* (B), compartment (C), and compartment and *Panicum* species (D). Ecotype in (A) and (C) includes both *P. hallii* and *P. virgatum* ecotypes.

**A**		
**Factor**	**Unweighted**	**Weighted**
Compartment	13.9 (***)	16.1 (***)
Site	5.3 (***)	6.3 (***)
Ecotype	—	—

**B**				
**Factor**	***Panicum virgatum***		***Panicum hallii***	
	**Unweighted**	**Weighted**	**Unweighted**	**Weighted**
Compartment	11.3 (***)	11.8 (***)	17.0 (***)	21.0 (***)
Site	3.6 (***)	3.7 (***)	7.3 (**)	11.9 (***)

**C**			
**Factor**	**Compartment**	**Unweighted**	**Weighted**
Site	Rhizosphere	9.5 (***)	10.9 (***)
Species			—	2.8 (*)
Ecotype			—	—
Site	Root endosphere	—	—
Species			—	—
Ecotype			—	—

**D**					
**Factor**	**Compartment**	***P. virgatum* [%]**		***P. hallii* [%]**	
		**Unweighted**	**Weighted**	**Unweighted**	**Weighted**
Site	Rhizosphere	9.6 (***)	13.0 (***)	15.7 (***)	24.4 (***)
	Root endosphere	4.5 (*)	—	—	—
Ecotype	Rhizosphere	6.1 (*)	7.3 (*)	—	—
	Root endosphere	—	—	—	—

Values reported are statistically significant: codes: “*” ~ p = 0.05, “**” 0.01, “***” 0.001. “—” denotes no significant correlation.

### Geochemical Analyses

Soils from PKL and BFL sites were analyzed at Texas A&M AgriLife Extension Soil, Water and Forage Testing Laboratory. Procedures for individual macro- and micronutrients were applied as stated on their website http://soiltesting.tamu.edu/webpages/swftlmethods1209.html: pH was determined according to Schofield and Taylor (1955); conductivity was measured following Rhoades (1982); soil nitrate-N was determined according to Norman (1965); plant available phosphorus, potassium, calcium, magnesium, sulfur, and sodium were extracted using the Mehlich III extractant and determined by ICP (Mehlich, 1978). Soil organic matter content was determined according to Schulte and Hopkins (1996).

## Results and Discussion

In this study, we investigated the belowground bacterial communities of switchgrass and its model relative, *Panicum hallii*. We analyzed the microbial communities retrieved from plant root compartments (rhizosphere = RS, root endosphere = RE) of two *P. virgatum* and two *P. hallii* ecotypes planted at two sites in Texas (Figure 1). Soil chemistries at Pickle Research Station (PKL) and Brackenridge Field Lab (BFL) sites showed differences with respect to N, P, C, K, Mg, S, and Na concentrations with more variability among PKL soils as shown in Supplementary Figure S1. The variability observed across PKL sandy loam soils may be partly due to the different soil texture compared to BFL soils, which had a higher clay proportion and hence higher degree of compaction (data not shown). The high degree of soil density at the BFL site may also explain why amplification and sequencing efforts for soil samples from this site failed repeatedly and resulted in either low number of sequences or very low alpha diversity. Similarly, we found fewer reads on average in BFL vs. PKL RS and RE samples (Supplementary Figure S2). Alpha diversity analysis across samples from different *Panicum* species, ecotypes, and planting sites showed that RS microbial communities are generally more complex than RE communities (Figures 2A,B), which matched our expectations based on previous findings in switchgrass (Singer et al., 2018) and other host plants (Caporaso et al., 2010; Coleman-Derr et al., 2016; Wagner et al., 2016b). As microorganisms have to overcome plant immune defense mechanisms to inhabit the endosphere compartment, this generally leads to reduced complexity in the microbial community compared to respective surface communities, such as found in rhizosphere and phyllosphere (Schulz et al., 2006). Microbial community diversity and structure were comparable between BFL and PKL RS samples (Figures 2A,B, 3), which suggests that BFL RS samples did not suffer from the same technical difficulties as BFL soil samples. For consistency, we decided to discard bulk soil sequence data from both sites and focused on the microbial communities in RS and RE. However, the impact of site on the rhizosphere communities suggests that plants had time to recruit from their destination soils and/or show remaining legacy communities from their sites of origin. The impact of switchgrass cultivation on soils has been previously described (He et al., 2017; Chen et al., 2019). Hence our analyses focus more on general bacterial trends among *P. virgatum* and *P. hallii* genotypes and do not claim to be representative of a *Panicum* grass grown from seed in native soils.

### Site Impacts Rhizosphere, but Not Root Endosphere Microbial Communities

**Figure 4:**
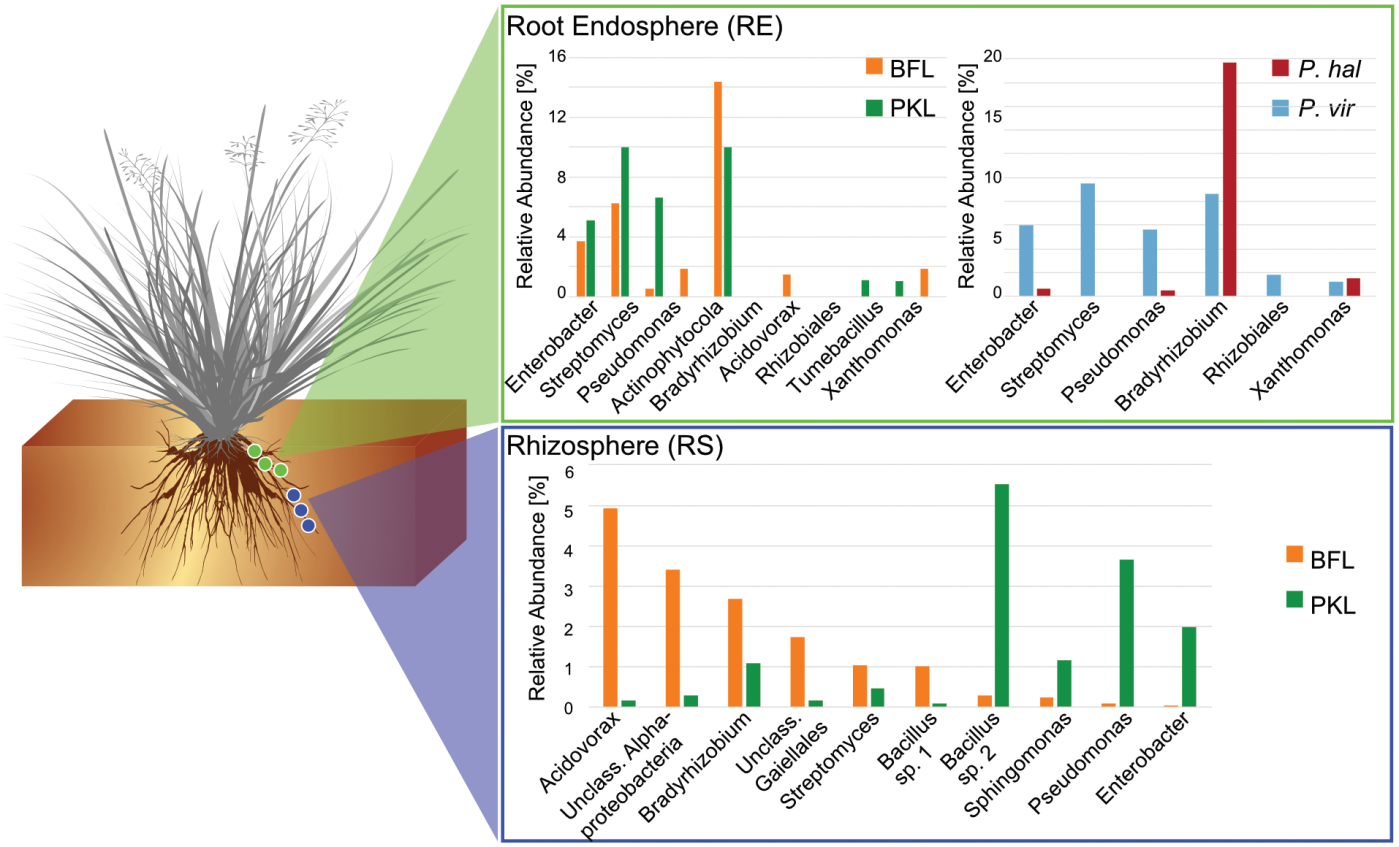
Comparison of core microbiomes in rhizosphere and root endosphere in *P. hallii* and *P. virgatum*. Relative abundances were determined separately for *P. hallii* and *P. virgatum* and for BFL and PKL sites, respectively. OTUs with relative abundances of >0.5% were included and classified to genus level were possible. The *Bacillus* genus is represented by two species.

Total bacterial community analysis of RS and RE samples showed a clear separation of RS samples by site, while RE communities did not display strong grouping by site (Figure 2C). Most abundant bacterial taxonomic groups in RS and RE included *Actinobacteria*, *Alpha-*, *Beta-*, and *Gammaproteobacteria* (Figure 4). We observed that bacterial community variability across all samples was affected by a combination of site, plant compartment, and plant genotype, which was independent of rarefaction (Tables 1A,B, Supplementary Tables S3A,B). These factors contributed relatively equally qualitatively (unweighted Unifrac) and quantitatively (weighted Unifrac) to microbial community composition and structure in RS and RE. Site explained ~10–11% of the community variability in the RS (Table 1C), which is in line with previous studies conducted across different geographical locations (Lundberg et al., 2012, 2013; de Menezes et al., 2014; Coleman-Derr et al., 2016; Fonseca-García et al., 2016; Yeoh et al., 2017). *Panicum* species impacted beta diversity only when considering relative (rarefied and non-rarefied) OTU abundances in the rhizosphere (Table 1C, Supplementary Table S3). The RE was not significantly impacted by site or host species but showed relatively small qualitative impact of *P. virgatum* genotype on its bacterial community (Tables 1C,D). For non-rarefied data, genotype did not show any significant impact on any of the studied bacterial communities (Supplementary Table S3). Overall, this indicates a relatively strong conservation of root endosphere microbial communities across the studied *Panicum* species. In the following, we will discuss core OTUs as well as OTUs that are unique to site, species, or genotype in the RS and RE.

Around 60 rhizosphere OTUs (97% identity) were recovered from 80% of all samples and were shared among *Panicum* species across both sites (Supplementary Table S5A) accounting for ~25% of the RS community. Both PKL and BFL RS showed comparable numbers of shared OTUs at all thresholds (Supplementary Table S5A), indicating that PKL RS microbial communities were more consistent than PKL soil nutrient concentration measurements and that processing of BFL RS samples did not result in biases potentially introduced due to the smaller grain size at BFL compared to PKL. The most abundant RS core OTUs present at both sites in *P. hallii* and *P. virgatum* belonged to the genera *Pseudomonas*, *Acidovorax*, *Bacillus*, and *Bradyrhizobium* (Figure 3, Supplementary Table S5B, Supplementary Figure S6). All of these genera encompass ubiquitous plant root-colonizing species, which have previously been reported in association with some symbiotic function (e.g., Höflich et al., 1994; Frey-Klett et al., 1997; Zablotowicz and Reddy, 2004). Although part of the overall RS core, the relative abundance patterns of each of the most abundant core OTUs varied largely between sites demonstrating that site did have a notable impact on the rhizosphere communities with respect to both the total community (Figure 2C) and the most abundant core OTUs (Figure 3). Several OTUs at >1% relative abundance showed significant abundance differences between PKL and BFL sites (Supplementary Table S4A) and just like the most abundant core microorganisms represented common plant- or soil-associated taxonomic groups, including *Bacillus*, *Pseudomonas*, *Enterobacter*, *Sphingomonas*, *Bradyrhizobium*, and *Streptomyces*. This demonstrates that planting site dramatically impacted the recruitment of rhizosphere microorganisms by the *Panicum* grasses. Total root endosphere bacterial communities were not significantly affected by site. Instead, the majority of RE core OTUs were shared between sites and will be discussed further in the following (Supplementary Table S5).

### *Panicum* Species Recruit Very Similar Root Microbiomes

We were interested in the types of taxa that are recruited by both *P. hallii* and *P. virgatum* roots. Identifying and quantifying the extent of overlapping core microorganisms and their relative abundance in the total communities is important in order to evaluate the suitability of *P. hallii* as a model for targeted switchgrass microbiome research, e.g., for the purpose of generating a synthetic microbial community and to conduct mechanistic studies with select microbial key players. In this context, we looked for common relative abundance and presence/absence patterns at the community and species level in *P. virgatum* and *P. hallii* RS and RE that would help us create hypotheses about similar recruitment strategies among these *Panicum* species that are, e.g., based on root exudate profiles and functional benefits to the plant. The RS was dominated by *Alpha*- and *Gammaproteobacteria*, and *Actinobacteria* and accounted for ~50% of the total RS bacterial community. There were no significant differences in relative abundance of OTUs belonging to these phyla/*proteobacterial* classes when comparing *P. virgatum* vs. *P. hallii* RS across both sites. The most abundant core RS OTUs shared among both sites and *Panicum* plants (Supplementary Table S5B) are mainly composed of *Acidovorax*, *Bacillus*, *Pseudomonas*, and unclassified *Alphaproteobacteria* (Figure 3, Supplementary Table S5B, Supplementary Figure S6). While these OTUs account for the most abundant core OTUs at either site independently of rarefaction, most of them show significant differences in relative abundance between sites (Figure 3, Supplementary Table S4A, Supplementary Figure S6). Generally, it appears that community variability driven by planting site and soil type mainly translates to quantitative differences among the most abundant RS core OTUs belonging to *Bacillus*, *Acidovorax* and unclassified *Alphaproteobacteria*, while less abundant OTUs appear relatively less important toward site specificity of the RS bacterial community. Rarefied and non-rarefied data tables agree with respect to the most abundant core OTUs and show comparable relative abundances (Figure 3, Supplementary Figure S6). The biggest difference is that there are slightly more core OTUs in the non-rarefied datasets (Supplementary Table S5A).

The RE bacterial communities differed slightly between BFL and PKL sites (Table 1D), but neither exhibited significant differences between *P. hallii* and *P. virgatum* species (Table 1C) nor between plant genotypes across both (Table 1C) or individually by *Panicum* species (Table 1D). Most dominant RE taxonomic groups included *Actinobacteria*, *Alpha-*, and *Gammaproteobacteria*. Compared to the RS, the RE bacterial community was significantly enriched in *Bradyrhizobium*, *Streptomyces*, and *Enterobacter*, which were also RE core OTUs (Supplementary Tables S4B, S5C). These RE core OTUs did not significantly vary between sites nor plant species. Comparison of core OTUs between PKL and BFL sites showed that 77.8% of all core OTUs were present at both sites and that relative abundances of the most abundant core OTUs *Bradyrhizobium*, *Streptomyces*, *and Enterobacter* are comparable (Figure 3). These three OTUs together accounted for ~17% and ~24% of the total communities at BFL and PKL, respectively. Comparison of RE core OTUs between *P. hallii* and *P. virgatum* showed that four of six core OTUs overlap between the plant species and all of the *P. hallii* core OTUs were also present in the *P. virgatum* RE core (Figure 3, Supplementary Figure S6). However, we noted differences in the community structures and observed that the *P. virgatum* core microbiome displayed more evenness, which was also affected by rarefaction (Figure 3, Supplementary Figure S6). Relative abundance analysis showed that the *P. hallii* RE core microbiome is dominated by *Bradyrhizobium* (19.7%), while the *P. virgatum* core OTU microbiome is more evenly represented by *Enterobacter*, *Bradyrhizobium*, *Streptomyces*, and *Pseudomonas* (Figure 3). The persistent occurrence and comparable relative abundance of *Bradyrhizobium* in the RE across sites and *Panicum* species, and independent of rarefaction, may suggest that members of this genus fulfill important functions for both switchgrass and *P. hallii*. Both *Panicum* species are known to thrive in marginal N-limited lands (Mitchell et al., 2016) and it can be speculated that this ability is in part due to symbiotic relationships that plants undergo with nitrogen-fixing, root-colonizing microbes (Roley et al., 2018, 2019). *Bradyrhizobium* is known for its ability to improve total plant nitrogen content via nitrogen fixation (Moulin et al., 2004; Hungria et al., 2015; Subramanian et al., 2015). Similarly, *Streptomyces* and *Pseudomonas* spp. have been observed to demonstrate plant growth-promoting effects via secondary metabolite production and nitrogen fixation, respectively (de Jesus Sousa and Olivares, 2016; Pham et al., 2017) and phosphate solubilization (Jog et al., 2014; Oteino et al., 2015). We hence speculate that these RE core OTUs were recruited into the *Panicum* RE because they could be involved in nutrient acquisition, systemic defense response, and other stress reduction, e.g., via salt tolerance. Most of the RE core OTUs also occurred in the RS core showing that recruitment of these OTUs is consistent occurring across field sites and that their presence is not necessarily related to vertical inheritance (Figure 3, Supplementary Tables S5B,C).

Our belowground analysis of *P. hallii* and *P. virgatum* rhizosphere and root endosphere at two sites shows that the studied *Panicum* species recruited similar RS and RE microbiomes. While overall bacterial community structures were not impacted by host species, we observed more granular differences among the dominant core RS and RE OTUs. While the RS OTUs showed significant statistical abundance differences between sites, it remains to be disclosed if their abundance differences are matched by relevant functional gene differences, which allow adaptation to different soil chemical and physical characteristics. Neither total RE bacterial communities nor core RE OTUs were significantly impacted by site or *Panicum* species. *Bradyrhizobium* appeared consistently across all included plants in this study and presents a target genus for functional studies in both switchgrass and *P. hallii*.

The microbiome comparison between various genotypes of *P. virgatum* and *P. hallii* in this study denotes a first step in better understanding common trends among *Panicum* bacterial communities. The apparent co-evolution of *Panicum* species and the significant overlap in endophytic bacterial communities between these two species, all four studied genotypes, and even between two sites with completely different soils is promising, for example for parallel field and laboratory investigations with switchgrass and *P. hallii*, respectively. *P. hallii*, which grows faster and thanks to its smaller biomass fits in controlled growth chambers, could be used to form hypotheses about environmental stress response, such as drought, increased temperature, nutrient limitation, soil freezing, and pathogen response, which in turn may be tested in field experiments with *P. hallii* and further with switchgrass. Microbial community responses under various growth conditions could hence be observed in higher throughput experiments using *P. hallii* than would be possible with *P. virgatum.* Aside from community diversity and structure investigations, microbial functional data retrieved via shotgun metagenome data and/or targeted isolate experiments linked to *Panicum* genetics and adaptation under conditions of stress could provide additional opportunities for advancing the successful use of switchgrass as a biofuel crop.

## Conclusions

This study presents the first comparison of rhizosphere and root endosphere microbiomes from *Panicum virgatum* and *Panicum hallii* upland and lowland genotypes. Our results suggest that there are significant similarities among all studied *Panicum* rhizosphere and root endosphere bacterial communities that together with *P. hallii*’s genetic features render *P. hallii* as a promising model plant for the exploration of *Panicum-*microbe-soil interaction studies.

## Data Availability Statement

The datasets generated for this study can be found at https://genome.jgi.doe.gov/portal/switchgrassmicrobiome.

## Author Contributions

ES, TW, and TJ designed the study and wrote the manuscript. ES and JB conducted field sampling. ES processed the samples, and generated and analyzed sequence data.

### Conflict of Interest

The authors declare that the research was conducted in the absence of any commercial or financial relationships that could be construed as a potential conflict of interest.

## Footnotes

^1^https://1ofdmq2n8tc36m6i46scovo2e-wpengine.netdna-ssl.com/wp-content/uploads/2019/07/iTag-Sample-Preparation-for-Illumina-Sequencing-SOP-v1.0.pdf, http://1ofdmq2n8tc36m6i46scovo2e-wpengine.netdna-ssl.com/wp-content/uploads/2017/09/iTag-Sample-Amplification-QC-v1.5.pdf
