# Supplementary material for: Conservation of Endophyte Bacterial Community Structure Across Two Panicum Grass Species
Source: Front Microbiol. 2019 Sep 27;10:2181. doi: 10.3389/fmicb.2019.02181 (PMC6777145; doi:10.3389/fmicb.2019.02181)
Supplement: Supplementary file 1 [file Table_1.DOCX]

**Supplementary Information**

**Table S1 | Environmental metadata for original sites from each genotype and planting site.** Genotypes AP13 and VS16 represent *Panicum virgatum*. *Panicum hallii* is represented by two variations, *P. hallii hallii* and *P. hallii filipes.*

| **Genotype** | **Site** | **Average T [°C]** | **Precipitation [mm]** |
| --- | --- | --- | --- |
| AP13 | Live Oak County, TX | 22 | 685 |
| VS16 | Nebraska City, NE | 17 | 856 |
| *Hallii hallii* | Wildflower Center, Austin, TX | 22 | 685 |
| *Hallii filipes* | Botanical Garden, Corpus Christy, TX | 22 | 685 |
| All | Brackenridge Field Lab, TX | 17 | 609 |
| All | Pickle Research Center, TX | 17 | 609 |

**Table S2 | Sample count overview.** BFL is abbreviated for Brackenridge Field Lab, PKL is short for Pickle Research Center. Summarized are samples that are represented by amplicon sequence data.

| **Site** | **Compartment** | ***Panicum* Species** | **Genotype** | **Sample count** |
| --- | --- | --- | --- | --- |
| BFL | Root Endosphere | *P. virgatum* | AP13 | 9 |
|  |  |  | VS16 | 7 |
|  |  | *P. hallii* | *hallii* | 3 |
|  |  |  | *filipes* | 4 |
|  | Rhizosphere | *P. virgatum* | AP13 | 14 |
|  |  |  | VS16 | 10 |
|  |  | *P. hallii* | *hallii* | 10 |
|  |  |  | *filipes* | 9 |
| PKL | Root Endosphere | *P. virgatum* | AP13 | 14 |
|  |  |  | VS16 | 15 |
|  |  | *P. hallii* | *hallii* | 4 |
|  |  |  | *filipes* | 5 |
|  | Rhizosphere | *P. virgatum* | AP13 | 17 |
|  |  |  | VS16 | 13 |
|  |  | *P. hallii* | *hallii* | 2 |
|  |  |  | *filipes* | 4 |
|  | Soil | |  | 17 |

**Table S3 |** **Percent bacterial/archaeal community variability according to weighted and unweighted Unifrac distance explained by factors for all samples (A), samples grouped by *Panicum species* (B), by compartment (C) and by compartment and *Panicum* species (D) for unrarefied datasets.** Values reported are statistically significant: codes: ‘*’ ~ p-value 0.05, ‘**’ 0.01, ‘***’ 0.001. ‘--‘ denotes no significant correlation.

A

| **Factor** | **unweighted** | **weighted** |
| --- | --- | --- |
| Compartment | 11.0*** | 15.7*** |
| Site | 3.7*** | 6.2*** |
| Genotype | -- | -- |
| Species | -- | -- |

B

| **Factor** | ***Panicum virgatum*** | | ***Panicum hallii*** | |
| --- | --- | --- | --- | --- |
|  | **unweighted** | **weighted** | **unweighted** | **weighted** |
| Compartment | 9.7** | 13.8*** | 14.7*** | 22.4*** |
| Site | 3.5** | 4.4*** | 6.9** | 15.7*** |

C

| **Factor** | **Compartment** | **unweighted** | **weighted** |
| --- | --- | --- | --- |
| Site | Rhizosphere | 8.3*** | 16.7*** |
| Species |  | -- | 3.1* |
| Genotype |  | -- | -- |
| Site | Root Endosphere | -- | -- |
| Species |  | -- | -- |
| Genotype |  | -- | -- |

D

| **Factor** | **Compartment** | ***P. virgatum*** **[%]** | | ***P. hallii* [%]** | |
| --- | --- | --- | --- | --- | --- |
|  |  | unweighted | weighted | unweighted | weighted |
| Site | Rhizosphere | 7.9 (***) | 15.2 (***) | 13.2 (**) | 24.4 (***) |
|  | Root Endosphere | 4.8 (*) | -- | -- | -- |
| Genotype | Rhizosphere | -- | -- | -- | -- |
|  | Root Endosphere | -- | -- | -- | -- |

**Table S4 | Overview of bacterial OTUs with significantly different relative abundance between compartments and host species/genotypes.** Listed are OTUs with an average of > 1% relative abundance at one site and respective test statistic values and FDR corrected *P* values. OTUs marked in bold denote core OTUs with significantly different relative abundance by site or *Panicum* species.

A

| **RS** | **Mean relative abundance** | | **χ^2^** | **FDR corr. *P*** |
| --- | --- | --- | --- | --- |
| **Genus** | **PKL** | **BFL** |  |  |
| ***Bacillus* (6)** | **5.5** | 0.3 | 19.4 | 2.9E-04 |
| ***Pseudomonas* (2)** | **3.7** | 0.1 | 8.4 | 2.5E-02 |
| ***Enterobacter* (0)** | **2.0** | 0.0 | 8.3 | 2.6E-02 |
| ***Sphingomonas* (34)** | **1.2** | 0.2 | 7.7 | 3.4E-02 |
| **Acidovorax (12)** | 0.2 | **4.9** | 12.6 | 4.7E-03 |
| **Unclass. *Alphaproteobacteria* (42)** | 0.3 | **3.4** | 37.7 | 2.4E-06 |
| ***Bradyrhizobium* (8)** | 1.1 | **2.7** | 9.6 | 1.5E-02 |
| ***Unclass. Gaiellales* (120)** | 0.2 | **1.7** | 37.4 | 2.4E-06 |
| ***Streptomyces*** **(1)** | 0.5 | **1.0** | 7.7 | 3.4E-02 |
| Unclass. *Actinobacteria* (219) | 0.4 | **1.0** | 22.7 | 9.3E-05 |
| ***Bacillus* (121)** | 0.1 | **1.0** | 26.5 | 2.7E-05 |

B

| **BFL & PKL** | **Mean relative abundance** | | **χ^2^** | **FDR corr. *P*** |
| --- | --- | --- | --- | --- |
| **Genus** | **RE** | **RS** |  |  |
| *Bradyrhizobium* | **11.8** | 2.0 | 18.7 | 1.2E-03 |
| *Streptomyces* | **8.5** | 0.8 | 20.7 | 5.6E-04 |
| *Enterobacter* | **4.5** | 0.9 | 10.0 | 4.3E-02 |
| *Pseudomonas* | **4.2** | 1.6 | 9.9 | 4.5E-02 |
| *Acidovorax* | 0.7 | **2.9** | 14.5 | 6.8E-03 |
| *Bacillus* | 0.2 | **2.6** | 41.0 | 1.3E-07 |
| Unclass. *Alphaproteobacteria* | 0.1 | **2.0** | 43.5 | 5.6E-08 |
| Unclass. *Gaiellales* | 0.0 | **1.1** | 49.0 | 5.2E-09 |

**Table S5 | Core microbiome statistics for *P. virgatum* and *P. hallii* genotypes** (A) by plant compartment across PKL and BFL sites represented as number of OTUs shared by % of samples; * denotes non-rarefied datasets for comparison. (B-D) enlist shared OTUs in 80% of all samples, relative abundances of each OTU were calculated for the respective compartment and site. Numbers in parentheses indicate OTU IDs to facilitate cross-category comparisons. Only shared OTUs with >1% and ≥ 0.5% relative abundance in the RS and RE, respectively, were listed; remaining OTUs were summarized as ‘Others’.

A

| **% of samples** | **RS** | | | **RE** | | | **RS*** | | | **RE*** | | |
| --- | --- | --- | --- | --- | --- | --- | --- | --- | --- | --- | --- | --- |
|  | **Total** | **PKL** | **BFL** | **Total** | **PKL** | **BFL** | **Total** | **PKL** | **BFL** | **Total** | **PKL** | **BFL** |
| **50** | 210 | 427 | 325 | 36 | 47 | 35 | 529 | 971 | 642 | 56 | 68 | 64 |
| **60** | 120 | 260 | 218 | 23 | 22 | 21 | 298 | 617 | 338 | 32 | 31 | 29 |
| **70** | 61 | 143 | 139 | 11 | 14 | 13 | 145 | 267 | 200 | 15 | 17 | 18 |
| **80** | **32** | **56** | **80** | **6** | **7** | **7** | **72** | **126** | **100** | **6** | **6** | **10** |
| **90** | 13 | 20 | 28 | 1 | 3 | 1 | 22 | 47 | 32 | 3 | 3 | 4 |
| **100** | 2 | 4 | 3 | 1 | 1 | 1 | 1 | 6 | 3 | 0 | 0 | 2 |

B

| **Total RS** | **[%]** | **BFL RS** | **[%]** | **PKL RS** | **[%]** |
| --- | --- | --- | --- | --- | --- |
| *Acidovorax* (12) | 2.9 | *Acidovorax* (12) | 4.9 | *Bacillus* (6) | 5.5 |
| *Bacillus* (6) | 2.6 | Unclass*.* *Alphaproteobacteria* (42) | 3.4 | *Pseudomonas* (2) | 3.7 |
| Unclass*.* *Alphaproteobacteria* (42) | 2.0 | ***Bradyrhizobium* (8)** | **2.7** | *Enterobacter* (0) | 2.0 |
| ***Bradyrhizobium* (8)** | **2.0** | *Unclass. Gaiellales* (120) | 1.7 | Sphingomonas (34) | 1.2 |
|  |  | *Streptomyces* (1) | 1.0 | ***Bradyrhizobium* (8)** | **1.1** |
|  |  | Bacillus (121) | 1.0 |  |  |
| Others (n=28) | 10.2 | Others (n=74) | 26.4 | Others (n=51) | 14.6 |

C

| **Total RE** | **[%]** | **BFL RE** | **[%]** | **PKL RE** | **[%]** |
| --- | --- | --- | --- | --- | --- |
| ***Streptomyces* (1)** | **7.7** | ***Actinosynnema* (8)** | ***5.3*** | ***Streptomyces* (1)** | ***9.2*** |
| ***Bradyrhizobium* (6)** | **6.5** | ***Bradyrhizobium* (6)** | **9** | ***Pseudomonas* (2)** | **8.9** |
| ***Pseudomonas* (2)** | **5.8** | ***Streptomyces* (1)** | ***4.8*** | ***Bradyrhizobium* (6)** | **6.6** |
| *Enterobacter* (0) | 4.4 | *Enterobacter* (0) | 3.5 | *Sphingobium* (14) | 3.9 |
| ***Actinosynnema* (8)** | **3.1** | ***Pseudomonas* (2)** | **1.8** | ***Actinosynnema* (8)** | ***1.1*** |
| ***Rhizobiales* (20)** | **1** | ***Rhizobiales* (20)** | **1.6** | ***Rhizobiales* (20)** | **0.9** |
| ***Bacillus* (9)** | **0.9** | *Xanthomonas* (216) | 1.2 | *Xanthomonas* (216) | 0.6 |
| ***Acidovorax* (12)** | **0.5** | *Ochrobactrum* (28) | 1.1 | *Frankiaceae* (46) | *0.4* |
| *Mycobacterium* (45) | 0.4 | ***Acidovorax* (12)** | **1.1** | Streptomyces (16) | *0.4* |
| *Frankiaceae* (46) | 0.4 | ***Bacillus* (9)** | **0.9** | ***Bacillus* (9)** | **0.3** |
|  |  | *Acidibacter* (55) | 0.3 | ***Acidovorax* (12)** | **0.3** |

**Figure S1 | Soil chemistry analysis of BFL and PKL sampling sites.** Comparison of individual chemicals show larger heterogeneity among samples at the PKL than the BFL site, which is likely due to the sandy nature of the PKL soil.

**Figure S2 | Number of reads per compartment and site before rarefaction.**

**Figure S3 | Number of samples per sample group in dependence of rarefaction depth.** Vertical line indicates rarefaction depth chosen at 10k sequences. RS = Rhizosphere, RE = Root Endosphere, FIL = *P. hallii filii,* HAL = *P. hallii hallii*.

B

A

C

**Figure S4 | Chao1 richness indicator as a function of rarefaction depth.** A) RE samples only, B) RS samples only up to 10k sequences, C) RS samples only up from 10k-20k sequences.

**Figure S5 | Shannon’s (*H*) index by compartment and genotype at PKL (A) and BFL (B) field sites.** RS = Rhizosphere, RE = Root Endosphere, FIL = *P. hallii filii,* HAL = *P. hallii hallii*.

**Figure S6 | Comparison of unrarefied core microbiomes in rhizosphere and root endosphere in *P. hallii* and *P. virgatum*.** Relative abundances were determined separately for *P. hallii* and *P. virgatum* and for BFL and PKL sites, respectively. OTUs with relative abundances of > 1.0% were included.
